# Prediction of long-term clinical outcomes using simple functional exercise performance tests in patients with COPD: a 5-year prospective cohort study

**DOI:** 10.1186/s12931-017-0598-6

**Published:** 2017-06-02

**Authors:** Sarah Crook, Anja Frei, Gerben ter Riet, Milo A. Puhan

**Affiliations:** 1 Epidemiology, Biostatistics and Prevention Institute, University of Zurich, Zurich, Switzerland; 2 Department of General Practice, Academic Medical Center, University of Amsterdam, Amsterdam, Netherlands

**Keywords:** COPD, Prediction, Mortality, Exacerbations, HRQoL, Longitudinal

## Abstract

The 1-min sit-to-stand (1-min STS) test and handgrip strength test have been proposed as simple tests of functional exercise performance in chronic obstructive pulmonary disease (COPD) patients. We assessed the long-term (5-year) predictive performance of the 1-min sit-to-stand and handgrip strength tests for mortality, health-related quality of life (HRQoL) and exacerbations in COPD patients. In 409 primary care patients, we found the 1-min STS test to be strongly associated with long-term morality (hazard ratio per 3 more repetitions: 0.81, 95% CI 0.65 to 0.86) and moderately associated with long-term HRQoL. Neither test was associated with exacerbations. Our results suggest that the 1-min STS test may be useful for assessing the health status and long-term prognosis of COPD patients. This study was registered at http://www.clinicaltrials.gov/ (NCT00706602, 25 June 2008).

## Background

The 1-min sit-to-stand (1-min STS) test and handgrip strength test have been proposed as simple tests of functional exercise performance in chronic obstructive pulmonary disease (COPD) patients [1, 2] that may make assessment of functional exercise performance in practice more accessible. Functional exercise performance is a known predictor of clinical outcomes in COPD [3]. In a cohort of primary care COPD patients (ICE COLD ERIC), we have previously found functional exercise performance measured with the 1-min STS test to be strongly associated with mortality, and both the 1-min STS and handgrip strength tests to be moderately associated with HRQoL, but not associated with exacerbations over 2 years of follow-up [2]. Now that data from the complete follow-up of the ICE COLD ERIC cohort is available, we aimed to further investigate the predictive properties of the 1-min STS and handgrip strength tests for long-term health outcomes over 5 years of follow-up.

## Methods

Four hundred nine COPD patients were recruited from primary care practices in Switzerland and the Netherlands and followed for up to 5 years. Full details of the study design and baseline characteristics of the cohort have been previously published [4, 5]. Patients were assessed with the 1-min STS test and handgrip strength test at baseline according to standardised protocols [2]. The outcomes of interest were mortality (exact dates of death retrieved from patients’ primary care practitioners), HRQoL (biannual assessments of the Chronic Respiratory Questionnaire (CRQ) [6]) and exacerbations (centrally adjudicated by independent experts using an event-based definition [7]).

We assessed the association of each test with mortality using cox-proportional hazards models, number of exacerbations with negative binomial regression models and HRQoL with multilevel linear regression models, each with one domain of the CRQ as the outcome and a random-intercept for time. All models were adjusted for appropriate confounders. Models were adjusted for age, sex, forced expiratory volume in 1 s (FEV_1_) L, CRQ dyspnoea and use of LABA/ICS, except the models with CRQ dyspnoea as the outcome, which were not adjusted for dyspnoea. The models for exacerbations were additionally adjusted for the number of exacerbations in the year before baseline. In the current analysis, we calculated estimates for handgrip strength per 1 kg and per 5 kg, and for the 1-min STS test per 1 repetition and per 3 repetitions, which has recently been suggested as the minimal important difference in COPD patients [8]. For mortality, we additionally calculated the area under the curve (AUC) for commonly used predictors and indices for mortality to compare their predictive discrimination abilities. All analyses were conducted using Stata version 14.1.

## Results

We included all 409 patients for the handgrip strength test and 371 patients for the 1-min STS test. Patients were excluded if they did not conduct the 1-min STS test at baseline (*n* = 5) or if they were not able to perform any repetitions in the 1-min STS test (*n* = 33). Baseline mean (standard deviation (SD)) age was 67.3 (10.0) and 57% were male. Median (interquartile range) FEV_1_ % of predicted at baseline was 58 (44, 68) and the mean (SD) CRQ dyspnoea score was 4.7 (1.6). The mean (SD) number of repetitions performed at baseline was 18.7 (8.3) and handgrip strength (kg) was 42.8 (10.1). After 5 years, 77 (19%) of patients had died and exacerbation rate of 0.8 per person-year with a total of 1031 exacerbations.

**Fig. 1:**
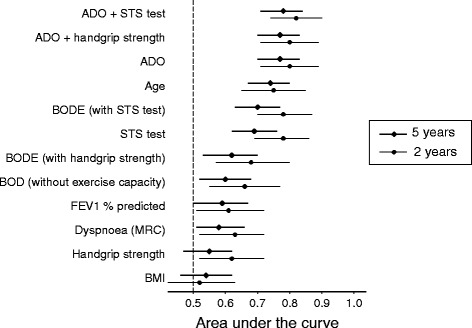
Areas under the *curve* and 95% confidence intervals for predictors of 5-year mortality compared to predictors of 2-year mortality in COPD patients. ADO, age, dyspnoea, airflow obstruction; STS, sit-to-stand; BMI, body mass index; BODE, BMI, airflow obstruction, dyspnoea, exercise capacity; MRC, Medical Research Council dyspnoea scale; FEV1, forced expiratory volume in 1 s

The results of the 5-year analyses for each functional exercise performance test and each outcome are presented in Table 1. The 1-min STS test was statistically significantly associated with 5-year mortality per 3 repetitions (hazard ratio (HR): 0.81, 95% CI 0.65 to 0.86), whereas the handgrip strength test was not statistically significantly associated with mortality. Both the 1-min STS test and handgrip strength test were moderately associated with HRQoL, and neither test was statistically significantly associated with exacerbations over 5 years. We performed a sensitivity analysis restricted to severe exacerbations (*n* = 112) for the 1-min STS test and the incidence rate ratio per 3 repetitions was 0.96 (95% CI 0.82 to 1.12). AUC values for both tests are shown in Fig. 1. After 5 years, the 1-min STS test alone still had a larger AUC for predicting mortality than other common individual predictors (FEV_1_ % predicted, dyspnoea and body mass index (BMI)), and was also the best performing modifiable individual predictor. The 1-min STS test alone performed better than the BODE index without an exercise component (BOD) and increased the prognostic value when added as the exercise component of BODE, but alone did not perform better than the ADO index (Fig. 1).

**Table 1:** Associations of the 1-min sit-to-stand (1-min STS) test and handgrip strength test with mortality, exacerbations and health related quality of life (HRQoL)

Outcome	1-min STS test (*n* = 371)	Handgrip strength test (*n* = 409)	
Mortality	Hazard ratio (95% CI)		Hazard ratio (95% CI)
Per 1 more rep	0.93 (0.89 to 0.97)	Per 1 more kg	0.97 (0.94 to 1.00)
Per 3 more reps	0.81 (0.65 to 0.86)	Per 5 more kg	0.86 (0.73 to 1.01)
Exacerbations	Incidence rate ratio (95% CI)		Incidence rate ratio (95% CI)
Per 1 more rep	1.00 (0.99 to 1.02)	Per 1 more kg	1.00 (0.98 to 1.02)
Per 3 more reps	1.01 (0.93 to 1.09)	Per 5 more kg	1.00 (0.92 to 1.08)
HRQoL	Effect (95% CI)		Effect (95% CI)
CRQ dyspnoea			
Per 1 more rep	0.05 (0.03 to 0.06)	Per 1 more kg	0.02 (−0.00 to 0.03)
Per 3 more reps	0.15 (0.16 to 0.32)	Per 5 more kg	0.08 (−0.00 to 0.15)
CRQ fatigue			
Per 1 more rep	0.03 (0.02 to 0.05)	Per 1 more kg	0.02 (0.01 to 0.04)
Per 3 more reps	0.10 (0.06 to 0.14)	Per 5 more kg	0.12 (0.06 to 0.18)
CRQ emotional function			
Per 1 more rep	0.01 (0.00 to 0.03)	Per 1 more kg	0.01 (−0.00 to 0.02)
Per 3 more reps	0.04 (0.00 to 0.08)	Per 5 more kg	0.03 (−0.02 to 0.09)
CRQ mastery			
Per 1 more rep	0.02 (0.01 to 0.03)	Per 1 more kg	0.01 (−0.00 to 0.02)
Per 3 more reps	0.05 (0.02 to 0.09)	Per 5 more kg	0.04 (−0.01 to 0.10)

All models were adjusted for age, sex, FEV_1_ L, CRQ dyspnoea and LABA/ICS, except the model with CRQ dyspnoea as the outcome, which was not adjusted for dyspnoea. The models for exacerbations were additionally adjusted for the number of exacerbations in the year before baseline. CRQ score is from 0–7, 0 = maximal impairment, 7 = no impairment.

*Abbreviations*: *1-min STS test* 1-min sit-to-stand test, *rep* repetition, *HRQoL* health-related quality of life, *CRQ* Chronic Respiratory Questionnaire, *CI* confidence interval

## Discussion

We found that over 5 years, the 1-min STS test was strongly associated with mortality and moderately associated with HRQoL. The handgrip strength test was not statistically significantly associated with mortality or HRQoL. Neither test was associated with exacerbations.

Overall, our results were comparable to the results of our earlier study with a shorter, 2-year follow-up [2]. The strong association between 1-min STS test performance and mortality was only slightly reduced per 1 more repetition (2-year HR 0.90, 95% CI 0.83 to 0.97 and 5-year HR 0.93, 95% CI 0.89 to 0.97). The associations of the 1-min STS test with exacerbations, domains of the CRQ and between the handgrip strength test and all outcomes were also slightly reduced after 5 years compared to 2 years. The present study suggests that the 1-min STS test has long-term predictive validity for mortality and that associations with outcomes only diminish slightly over 5 years. However, the handgrip strength test loses its statistical association with mortality after a longer follow-up. This could be attributable to the different requirements of the two tests; the 1-min STS test is more physically demanding than the handgrip strength test and is thus likely to capture a better approximation of functional exercise performance.

The handgrip strength test has been proposed as a simple test for predicting all-course mortality across healthcare settings [9, 10]. Our results indicate that the 1-min STS test is an even more attractive test since it was a much stronger predictor for mortality than the hand-grip strength test (AUC for 1-min STS test 0.69 and handgrip-strength 0.55) and does not need expensive equipment, like a hand dynamometer. However, one limitation is that a small proportion of patients may not be able to conduct the 1-min STS test due to musculoskeletal or neurological limitations, which can be a common limitation with many functional exercise performance tests.

The lack of association seen between both tests and exacerbations suggests that they do not predict the incidence of exacerbations. In the sensitivity analysis restricted to severe exacerbations no association with the 1-min STS test was found, however the small number of severe exacerbations limits this analysis, and should be tested in patients with more severe COPD to confirm these results. Compared to the analysis at 2-years, we chose to include slightly different confounders in order to ensure a more comprehensive control of potential confounding. We therefore performed a sensitivity analysis using the original confounders to check how this change in methodology would impact the results. The results for all outcomes at 5 years using the original 2-year confounders were similar to the results with the updated confounders. We believe that the results found in our study are generalisable due to the large and diverse population in our cohort, which reflects a representative sample of COPD patients in primary care.

## Conclusion

In conclusion, the 1-min STS test as a measure of functional exercise performance showed good long-term predictive validity for the mortality and moderate predictive validity for HRQoL. This supports and extends the existing evidence that the 1-min STS test could be useful for assessing health status and long-term prognosis in COPD patients.

## Abbreviations

1-min STS: 1-minute sit-to-stand
AUC: Area under the curve
BMI: Body mass index
CI: Confidence interval
COPD: Chronic obstructive pulmonary disease
CRQ: Chronic respiratory questionnaire
FEV_1_: Forced expiratory volume in 1 s
HR: Hazard ratio
HRQoL: Health-related quality of life
SD: Standard deviation
